# Channel selection of metagenomic next-generation sequencing in infants pathogen detection: a multicenter cross-sectional study

**DOI:** 10.3389/fped.2025.1632123

**Published:** 2025-11-21

**Authors:** Chengcheng Yang, Minxu Li, Shumei Yang, Jianer Pan, Yue Ding, Jie Yang

**Affiliations:** 1 Department of Neonatology, Nanfang Hospital, Southern Medical University, Guangzhou, China; 2 Department of Neonatology, Dongguan Women and Children Hospital, Dongguan, Guangdong, China; 3 Department of Neonatology, Guangdong Women and Children Hospital, Guangzhou, China; 4 Department of Neonatology, Jiangmen Women and Children Hospital, Jiangmen, Guangdong, China

**Keywords:** neonate, infectious diseases, MNGs, channel, pathogene

## Abstract

In the neonatal period, infectious diseases associated with high morbidity (e.g., neonatal sepsis and meningitis) are preliminarily assessed using indicators like C-reactive protein (CRP) and procalcitonin, but definitive diagnosis relies on pathogen detection through methods such as blood culture, which is time-consuming and has low sensitivity. To improve diagnostic efficiency, metagenomic next-generation sequencing (mNGS) is increasingly utilized, offering three testing modalities: DNA-only, RNA-only, and combined DNA+RNA channels. This retrospective study analyzed 894 clinical samples (peripheral blood, sputum, bronchoalveolar lavage fluid) to compare detection rates across channels. The overall mNGS positivity rate was 51.9% (464/894), with no significant differences among DNA-only (50.8%), RNA-only (55.7%), and combined channels (49.6%) (*p* > 0.05). Notably, bronchoalveolar lavage fluid samples exhibited the highest positivity rate (84.57%, 148/175), reaching 97.33% (73/75) with dual-channel testing. Sputum samples showed a 53.7% positivity rate (87/162), increasing to 82.35% (14/17) with dual-channel detection. Conversely, peripheral blood had an overall positivity rate of 43.14% (132/306), with the DNA-only channel outperforming RNA-only and dual channels (45.34% 5s. 43.00% and 34.21%). These findings underscore the importance of channel selection based on sample type to optimize diagnostic accuracy and cost-effectiveness.

## 1 Background

Neonatal death remains the leading cause of mortality in children under five years of age, with infectious diseases accounting for a significant proportion of these deaths (1). Newborns are particularly vulnerable to infectious diseases due to their immature immune systems and potential vertical transmission of pathogens from the mother (2). In China, preterm and very low birth weight infants (VLBW) represent approximately 10% of live births (3). These infants face elevated risks of sepsis due to immunological immaturity and are prone to intracranial infections owing to a weakened blood-brain barrier. Such infections are associated with high mortality rates and poor long-term neurodevelopmental outcomes (4–6). Current diagnostic methods for neonatal infections primarily rely on nonspecific biomarkers, such as white blood cell count, CRP, and procalcitonin, which only indicate the possibility of infection (7–9). Furthermore, obtaining adequate clinical specimens from newborns for pathogen detection is challenging, particularly for blood volume-dependent tests like blood culture—the current gold standard for sepsis diagnosis. However, blood cultures are time-consuming and exhibit low sensitivity in neonatal sepsis (10), specially given their limited blood volume, which further reduces the detection rate compared to older children and adults (11).

mNGS is a laboratory diagnostic technology based on high-throughput sequencing technology to sequence the whole biological genome in a variety of clinical samples. By simultaneously detecting millions of DNA/RNA fragments, mNGS offers rapid and efficient pathogen detection, leading to its increasing adoption in clinical settings (12–15). Its clinical utility is particularly evident in identifying pathogens undetectable by conventional methods, though cost remains a limiting factor. When mNGS testing is selected, three sequencing modalities must be chosen: DNA-only, RNA-only, or combined DNA/RNA. The diagnostic performance of mNGS, as reflected by positivity rates, depends critically on this sequencing modalities selection. While multiple studies have validated mNGS efficacy in neonates (16–18) and Chinese guidelines outline its use in infant infections (19), real-world data on sequencing modality selection patterns and their impact on pathogen detection rates in this population remain rare. To address this gap, we conducted a multicenter, cross-sectional study to assess mNGS sequencing modality selection practices and their correlation with positivity rates and pathogen profiles in neonates.

## 2 Methodology

This multicenter cross-sectional study analyzed real-world data from five hospitals in China: Nanfang Hospital of Southern Medical University, Dongguan Women and Children Hospital, Guangdong Maternal and Child Health Hospital, Jiangmen Women and Children Hospital, and Lianjiang People's Hospital. All participating institutions met the following criteria: 1) annual delivery volume ≥5,000 births, and 2) availability of Level IV neonatal intensive care units (NICUs). We retrospectively analyzed neonates undergoing mNGS testing between January 2020 and December 2022. Testing indications followed national clinical guidelines (19).

Inclusion criteria: requiring both: a) clinical signs of infection, and b) negative conventional microbiological cultures. Exclusion criteria: a) Pathogens were detected by conventional microbiological tests, b) major congenital anomalies or chromosomal abnormalities, c) inadequate sample quality (e.g., hemolysis, insufficient volume, improper storage). Clinical data were extracted from the hospitals' electronic health records. The study protocol was approved by the Ethics Committee of Nanfang Hospital, Southern Medical University (Approval No. NFEC-2022-446).

mNGS employs a standardized clinical laboratory workflow comprising specimen processing; nucleic acid extraction (simultaneous DNA/RNA isolation); library preparation through fragmentation and adapter ligation; high-throughput sequencing on platforms; and bioinformatic analysis via alignment to microbial reference genomes and clinical pathogen databases (All nucleic acid samples were processed under a standardized protocol across all participating hospitals to ensure consistency and reproducibility. Specifically, RNA preservation was prioritized immediately after sample collection using RNase inhibitors and appropriate storage conditions to prevent degradation. The integrity of RNA was quantitatively confirmed prior to analysis via spectrophotometry or automated electrophoresis) (20).

Clinical specimens were collected from all enrolled patients for mNGS, with testing modality allocation (DNA-only, RNA-only, or combined DNA/RNA) determined by clinical indications. All samples underwent standardized collection and processing protocols to ensure analytical consistency.

### 2.1 Statistical analysis

All data in the study were statistically analyzed by SPSS 25.0. Count data is expressed as composition ratios or percentages (%), while measurement data is represented as mean ± standard deviation (mean ± SD). The differences in proportions across multiple groups were analyzed using Pearson's chi-square test. Results were considered statistically significant if *p* value was <0.05.

## 3 Results

### 3.1 Clinical characterization of the patients for inspection

A total of five hospitals participated in the study, including 509 patients (refer to Table 1). The total number of samples sent for testing was 894, with 464 positive cases detected, resulting in an overall positive rate of 51.90% (464/984).

**Table 1:** Clinical characterization of patients undergoing mNGS testing (*n* = 509).

Clinical characteristics	
Age (d)	28.46 ± 26.78
Gender (Male/Female)	320 (62.87%)/189 (37.13%)
Clinical diagnosis (number of cases):	
Pneumonia	51 (8.84%)
Intracranial infection	42 (8.25%)
Sepsis	26 (5.11%)
RDS	12 (2.36%)
Clinical manifestations: (number of cases)	
Fever	99 (36.5%)
Cough	50 (18.5%)
Abnormal white blood cells	68 (25.1%)

Statement: The clinical diagnosis was the primary one in this patient.

RDS, respiratory distress syndrome.

### 3.2 Selection of mNGS sequencing modalitiess and positive rates

**Figure 1:**
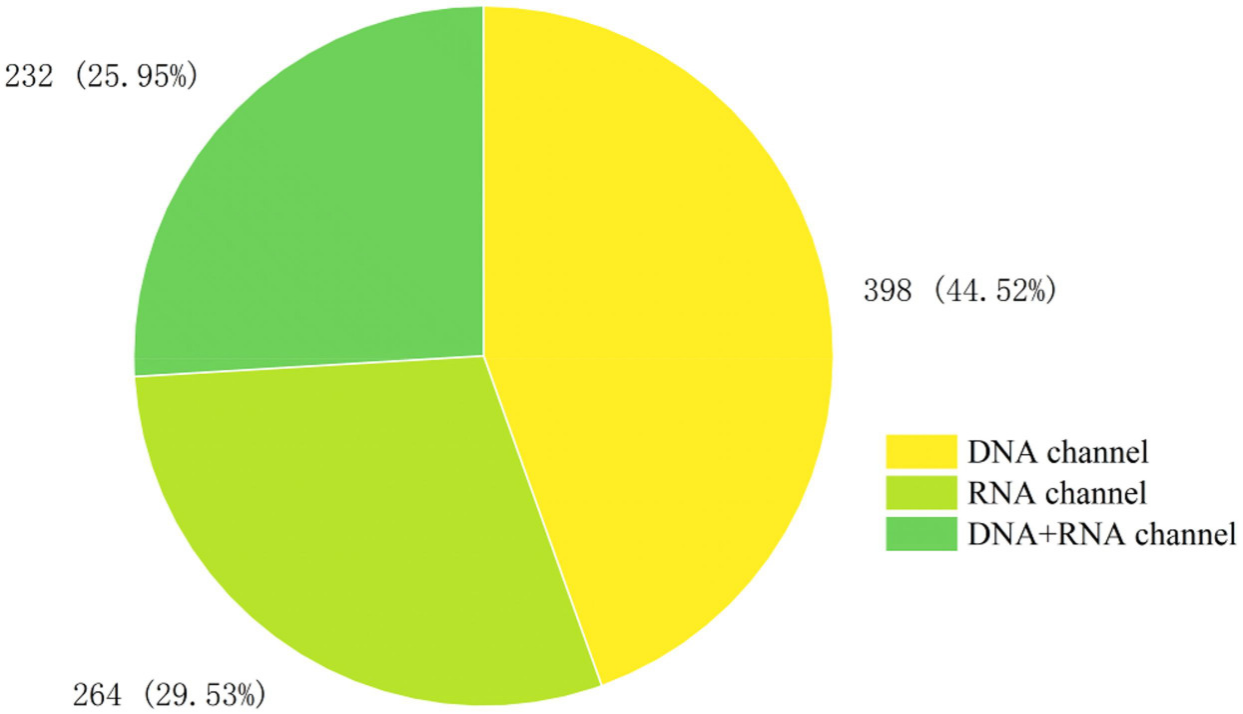
The inspection status of various channels.

A total of 894 clinical specimens were analyzed, with sequencing modalities distribution as follows (Figure 1): DNA-only (44.52%, 398/894), RNA-only (29.53%, 264/894), and combined DNA/RNA (25.95%, 232/894). The overall pathogen detection rate was 51.90% (464/894). Positivity rates varied marginally across modalities: RNA-only (55.68%, 147/264), DNA-only (50.75%, 202/398), and combined DNA/RNA (49.56%, 115/232). No statistically significant differences in positivity rates were observed among the three sequencing modalities (*p* > 0.05).

### 3.3 The proportion and positive rate of mNGS specimens

**Figure 2:**
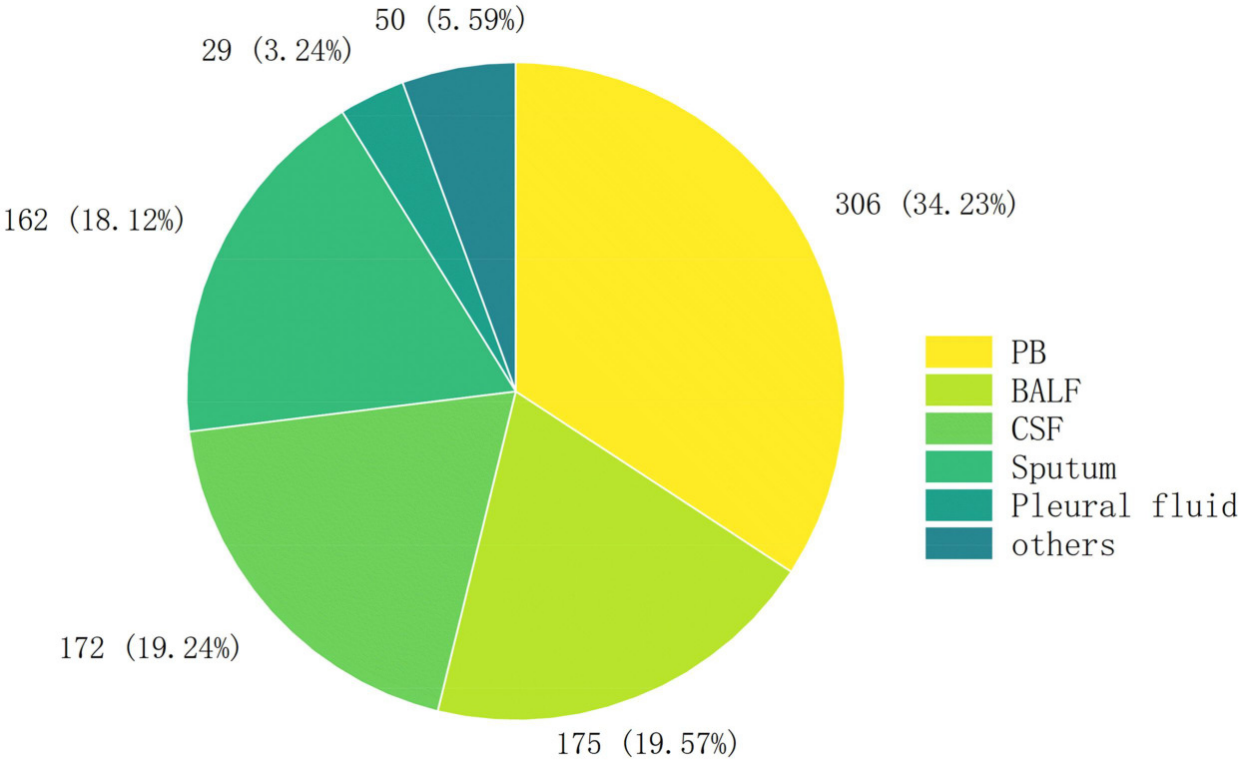
The number of cases in each sample.

**Figure 3:**
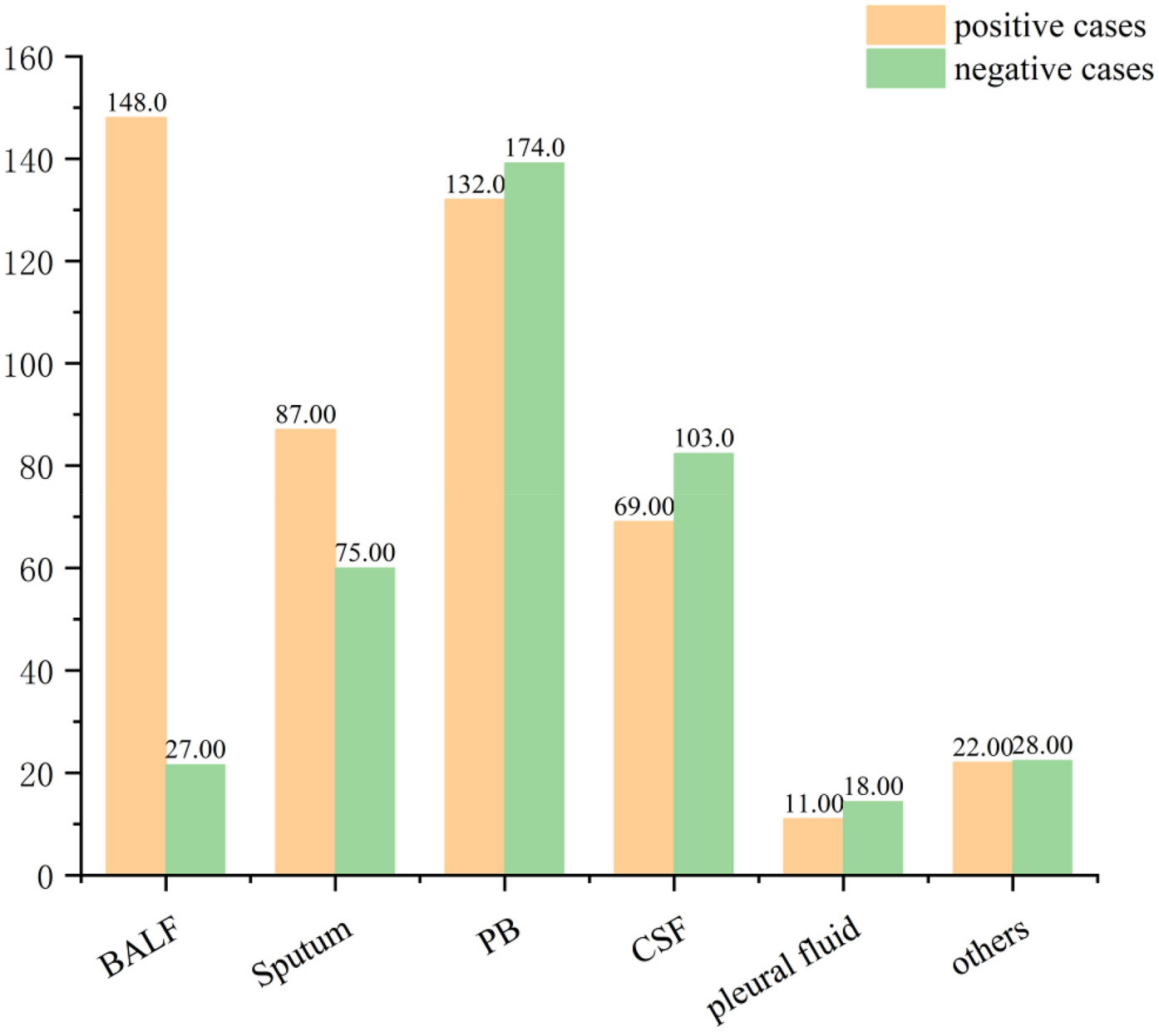
The Number of positive cases in each sample.

A total of 894 clinical specimens were analyzed (Figure 2), comprising peripheral blood (34.23%, 306/894), bronchoalveolar lavage fluid (BALF) (19.57%, 175/894), cerebrospinal fluid (19.24%, 172/894), and sputum (18.12%, 162/894). Rare specimen types (e.g., pleural effusion) constituted the remainder. BALF specimens exhibited the highest pathogen positivity rate (84.57%, 148/175), consistent with prior studies (21–23). followed by sputum (53.7%, 87/162), peripheral blood (43.14%, 132/306), and cerebrospinal fluid (40.12%, 69/172) (Figure 3).

#### 3.3.1 Different specimens and positive rates under different sequencing modalities

**Figure 4:**
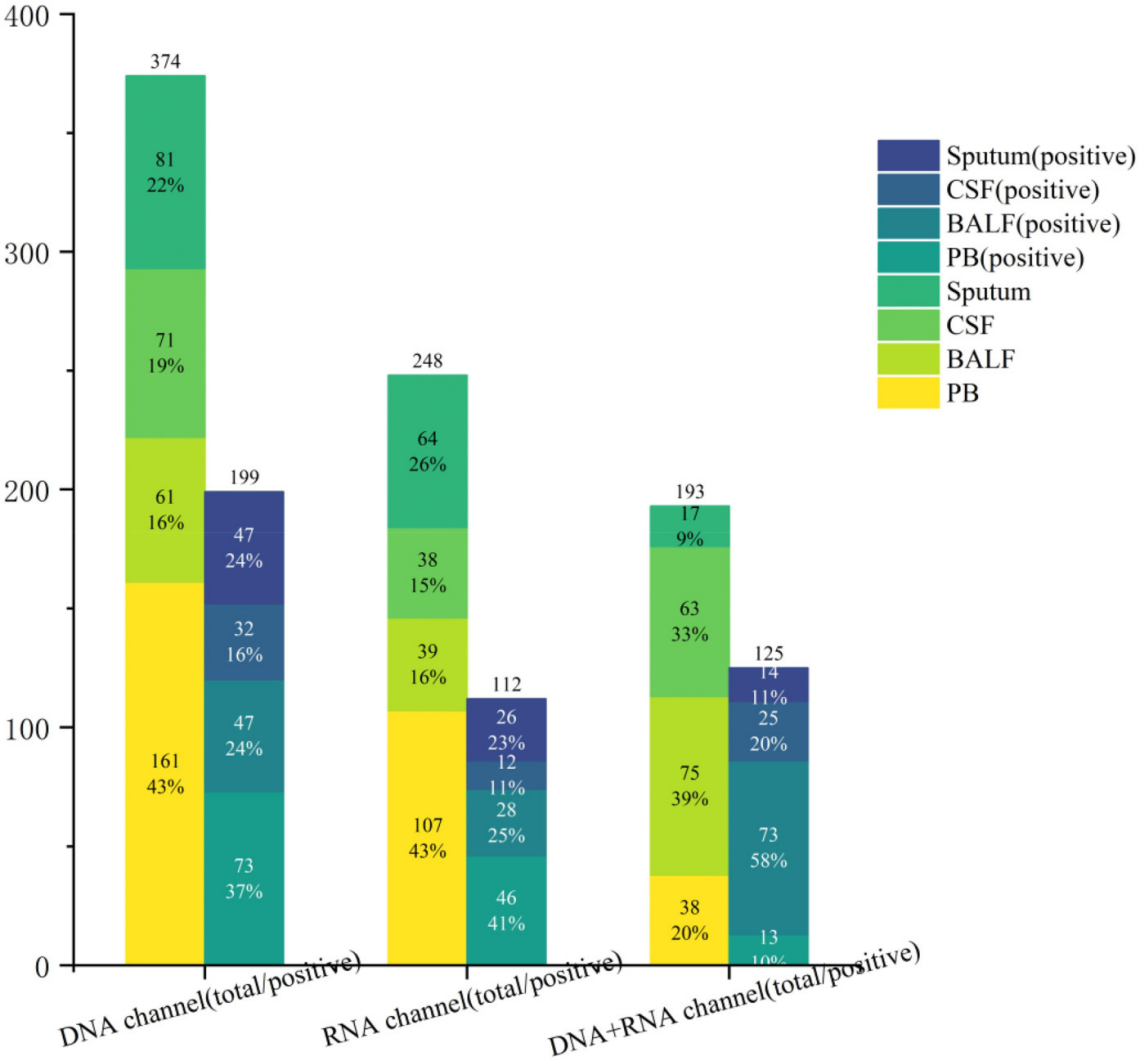
The number of positive cases for different samples under different channels.

We further compared pathogen detection rates across sequencing modalities for four specimen types (Figure 4). Among peripheral blood specimens (*n* = 306), DNA-only sequencing modality was predominantly utilized (56.86%, 161/306), demonstrating positivity rates of 45.34% (73/161) for DNA-only, 43.00% (46/107) for RNA-only, and 34.21% (13/38) for combined sequencing modalities, with no statistically significant inter-sequencing modalities differences (*p* = 0.460). In contrast, BALF specimens (*n* = 175) exhibited markedly enhanced diagnostic performance with combined DNA/RNA sequencing modalities, achieving a 97.33% positivity rate (73/75) compared to 77.05% (47/61) for DNA-only and 71.80% (28/39) for RNA-only sequencing modality (*p* < 0.05). Notably, despite its superior efficacy, combined-sequencing modalities accounted for only 42.86% (75/175) of BALF submissions, likely reflecting clinical hesitancy toward multi-sequencing modalities due to cost considerations or procedural priorities. Cerebrospinal fluid analysis (*n* = 172) revealed DNA-only sequencing modality as the most utilized modality (41.28%, 71/172), though positivity rates showed no significant inter-sequencing modalities variation: 45.07% (32/71) for DNA-only, 31.60% (12/38) for RNA-only, and 39.70% (25/63) for combined sequencing modalities (*p* = 0.390). In contrast, sputum specimens (*n* = 162) demonstrated profound combined-sequencing modalities superiority, achieving an 82.35% positivity rate (14/17) vs. DNA-only (58.02%, 47/81) and RNA-only (40.62%, 26/64) (*p* = 0.005). Notably, despite this performance advantage, combined-sequencing modalities accounted for only 10.49% (17/162) of sputum analyses, suggesting underutilization potentially driven by cost constraints or specimen prioritization protocols. The substantial cost disparity between combined-sequencing modalities (¥8,000) and DNA-only testing (¥1,950) at our institution—a 4.1-fold difference—likely contributed to the underutilization of combined modalities, despite their 23.33% absolute improvement in sensitivity for sputum specimens. These findings demonstrate: 1) DNA-sequencing modality preference for blood/CSF specimens correlates with marginally higher positivity rates; 2) Combined DNA/RNA sequencing modalities significantly enhance respiratory pathogen detection in BALF (97.33%) and sputum (82.35%); 3) Clinical practice patterns do not consistently align with optimal detection modalities, particularly for respiratory samples.

#### 3.3.2 Positive detection rates and specimens under different diagnoses

Among 51 neonates with clinically diagnosed pneumonia, mNGS identified pathogens in 76.47% of cases (39/51). BALF was the most submitted specimen (35.29%, 18/51), yielding the highest positivity rate (88.89%, 16/18). Sputum samples (11.76%, 6/51) showed a 66.67% positivity rate (4/6). Concurrent blood-sputum testing (25.49%, 13/51) demonstrated 61.54% positivity rate (8/13). While combined blood and BALF analysis (9.80%, 5/51) achieved 60.00% (3/5). Among intracranial infection cases (*n* = 42), cerebrospinal fluid analysis represented the predominant diagnostic approach (97.62%, 41/42), yielding a 51.22% detection rate (21/41). Notably, one complex case required concurrent analysis of blood, cerebrospinal fluid, and sputum specimens through multi-sequencing to identify the etiological agent. Among 27 sepsis cases, mNGS achieved a pathogen detection rate of 70.37% (20/27). Peripheral blood testing alone (33.33%, 9/27) yielded a positivity rate of 66.67% (6/9). Combined peripheral blood and sputum analysis (37.00%, 10/27) demonstrated the highest detection rate at 80.0% (8/10), while cerebrospinal fluid specimens (11.11%, 3/27) showed 66.67% positivity rate (2/3). Notably, concurrent testing of peripheral blood and cerebrospinal fluid identified adenovirus in one case. Given sepsis-associated rapid clinical deterioration and frequent central nervous system complications, empirical concurrent blood and cerebrospinal fluid testing is strongly recommended.

### 3.4 Detection of pathogens in total sample

**Figure 5:**
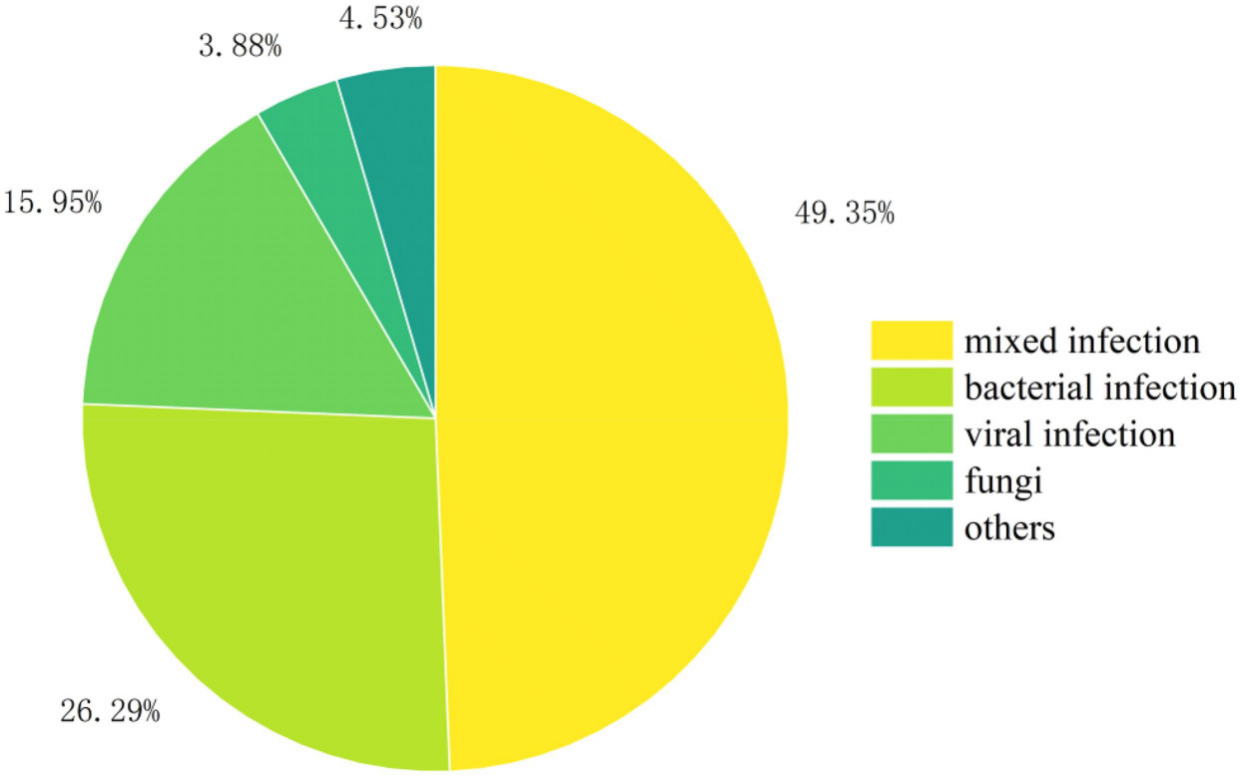
The distribution of pathogens in the overall sample.

Among 464 mNGS-positive cases, polymicrobial infections (≥2 pathogens) accounted for 49.35% (229/464), with single-pathogen infections constituting 50.65% (235/464). Bacterial pathogens predominated in monomicrobial infections (51.91%, 122/235), followed by viruses (31.49%, 74/235) (Figure 5). *Human herpesvirus 5* (HHV-5) emerged as the most prevalent pathogen (19.83%, 92/464), a critical finding given its high mortality in neonatal CNS infections (24). Major bacterial isolates included *Staphylococcus aureus* (10.34%, 48/464), *Klebsiella pneumoniae* (9.70%, 45/464), *Chlamydia trachomatis* (8.41%, 39/464), and *Escherichia coli* (8.19%, 38/464), all associated with severe outcomes—particularly drug-resistant strains causing meningitis or sepsis with poor neurological prognoses (25–27). Fungal pathogens (8.62%, 40/464) primarily involved *Pneumocystis jirovecii* (4.09%, 19/464), *Candida parapsilosis* (2.59%, 12/464), and *Candida albicans* (2.16%, 10/464), often secondary to empirical antibiotic use. While pulmonary/gastrointestinal fungal infections were common, disseminated cases (e.g., fungemia, cerebral involvement) carried significant mortality (28). Rapid mNGS-guided pathogen identification enables targeted antimicrobial therapy, potentially mitigating disease progression. When conventional diagnostics fail, empirical regimens should prioritize regionally prevalent pathogens identified through mNGS surveillance (29). (The detailed distribution of pathogens is presented in Table 2).

**Table 2:** The distribution of pathogens in the overall sample.

Pathogens (≥5)	Count
Virus:	
Human herpesvirus 5	92
Respiratory syncytial virus	32
Rhinovirus	14
Adenovirus	10
Bacteria:	
G+:	
Staphylococcus aureus	48
Streptococcus pneumoniae	36
Streptococcus agalactiae	18
Enterococcus faecium	13
Enterococcus faecalis	12
Mycobacterium tuberculosis	5
G-:	
Klebsiella pneumoniae	45
Escherichia coli	38
Acinetobacter baumannii	30
Pseudomonas aeruginosa	25
Haemophilus influenzae	19
Fungi:	
Pneumocystis jirovecii	18
Candida parapsilosis	12
Candida albicans	10
Aspergillus fumigatus	8
Ureaplasma:	
Ureaplasma urealyticum	16

Statement: This statistics is calculated based on the frequency of pathogen detection, including pathogens in mixed infections.

### 3.5 Detection of pathogens in different sequencing modalities

**Figure 6:**
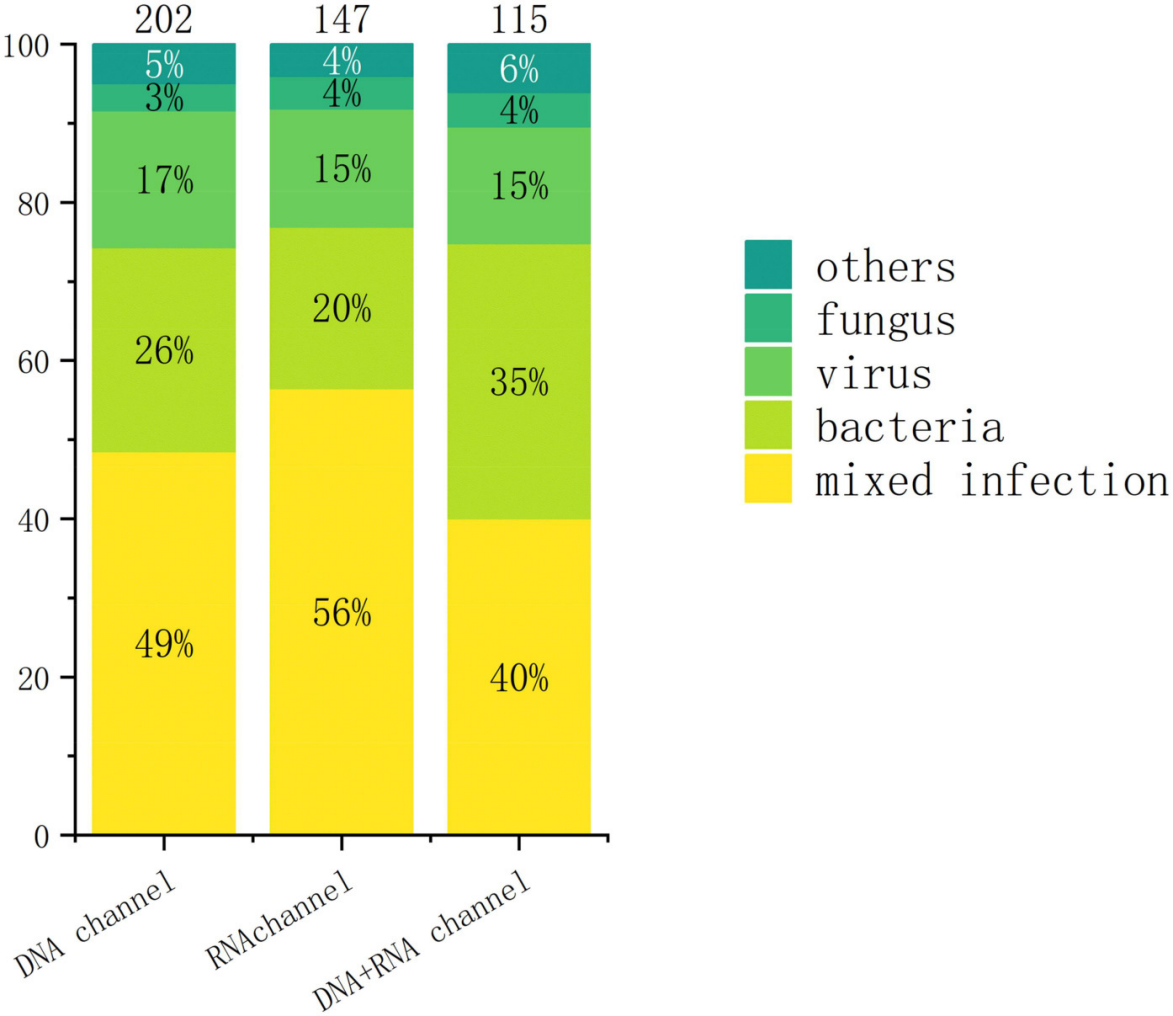
Distribution of pathogens in different channels.

**Figure 7:**
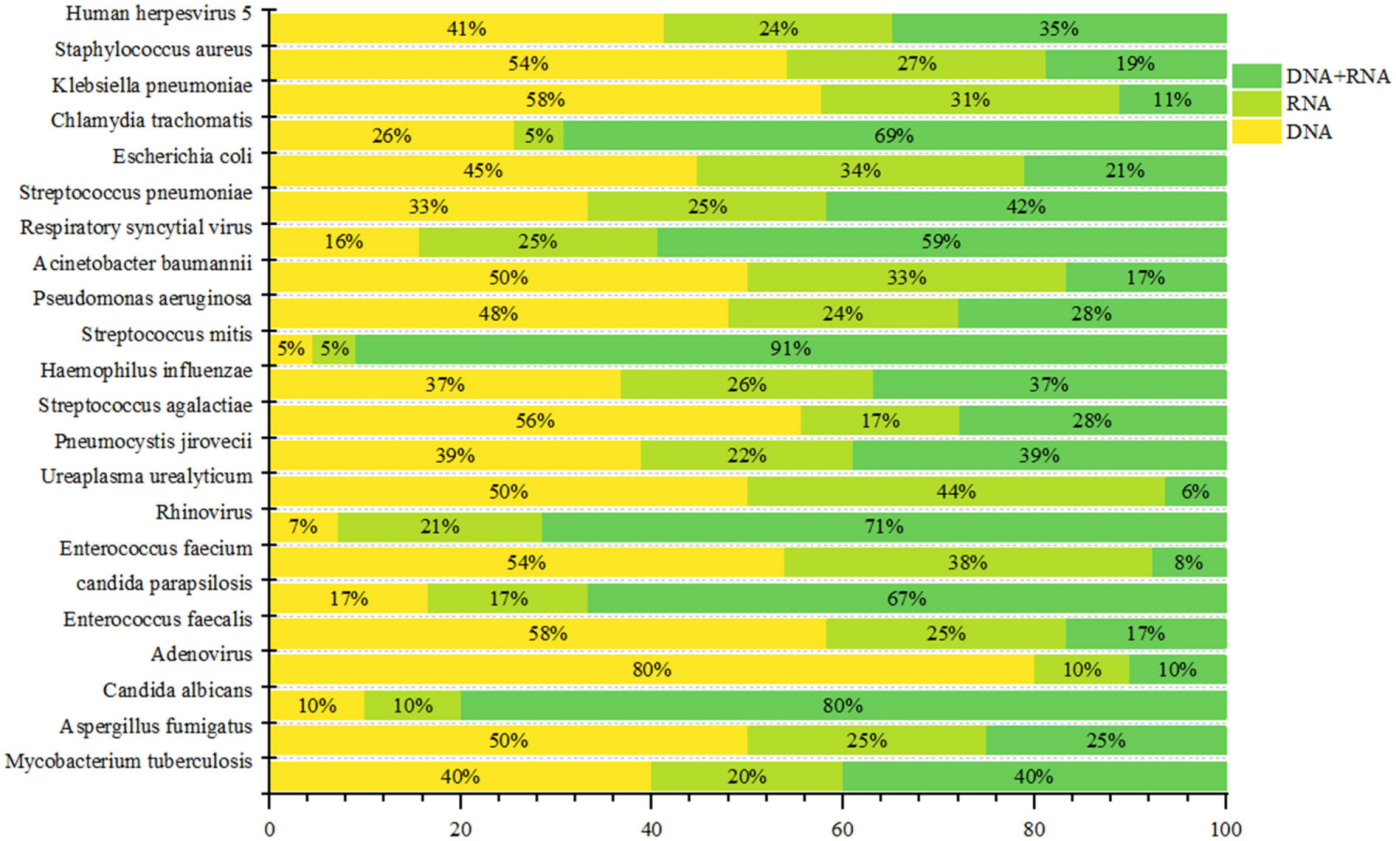
Distribution of pathogens in different channels (details).

Polymicrobial infections were predominant across all sequencing modalities, detected in 49.0% (98/200) of DNA-only, 56.00% (83/147) of RNA-only, and 40.00% (46/115) of combined DNA/RNA specimens. Monomicrobial infection profiles varied significantly by modality: DNA-only sequencing modality identified bacterial (26.00%, 52/200), viral (17.50%, 35/200), and fungal (3.50%, 7/200) pathogens; RNA-only sequencing modality detected bacterial (20.40%, 30/147), viral (15.00%, 22/147), and fungal (4.10%, 6/147) agents; while combined sequencing modalities revealed bacterial (34.80%, 40/115), viral (14.80%, 17/115), and fungal (4.30%, 5/115) targets (Figure 6). *Human herpesvirus 5* was the predominant pathogen in DNA-only and RNA-only sequencing modalitiess, whereas *Chlamydia trachomatis* prevailed in combined sequencing modalitiess (Figure 7). Bacterial detection via RNA sequencing modalitiess—despite DNA being their genetic material—may arise from high ribosomal RNA (rRNA) content during active transcription, as observed in PCR-based RNA assays (30), or detection of mRNA. Clinical validation remains critical to exclude false positives from contamination or technical artifacts (31).

## 4 Discussion

The application of mNGS in neonatal infections is becoming increasingly widespread. Notably, mNGS significantly enhanced central nervous system infection diagnosis compared to conventional methods (32). Conventional cultures showed markedly lower sensitivity: neonatal sepsis blood cultures (11), BALF cultures, and sputum cultures (33), with cerebrospinal fluid cultures performing even poorer (34). Additionally, conventional methods require prolonged turnaround times (days to weeks). These data suggest mNGS demonstrates superior diagnostic efficacy for respiratory pathogens, particularly in BALF. However, BALF acquisition via bronchoscopy limits its clinical feasibility. Sputum emerges as a practical alternative, offering a relatively high mNGS positivity rate. For neonatal sepsis, mNGS of peripheral blood showed a pathogen detection rate of 43.14% (132/306), outperforming conventional blood cultures which showed <10% sensitivity.

he selection of specimens undoubtedly influences the positivity rate of diagnostic tests. In clinical practice, the choice of sample type is primarily guided by the patient's clinical manifestations and their compliance with the procedure. For instance, in cases of systemic infection, peripheral blood is prioritized for testing. In patients presenting with convulsions or other central neurological symptoms, cerebrospinal fluid (CSF) is collected in addition to peripheral blood. For individuals with respiratory symptoms, suitable samples include sputum, throat swabs, or BALF. Among these, sputum and throat swabs are often preferred due to their non-invasive nature and ease of collection. In contrast, BALF requires an invasive procedure under anesthesia, which may not be acceptable to all patients or their families. We acknowledge that submitting multiple sample types for testing is theoretically ideal. However, this approach is often impractical in the context of clinical practice in China. Collecting various specimens from a pediatric patient within a short timeframe may raise concerns among family members, who could perceive it as overtreatment. Furthermore, repeated peripheral blood draws, in particular, may lead to non-compliance from the child patient.

Virtually all bacterial genetic material is composed of DNA. Standard DNA extraction protocols efficiently isolate bacterial DNA, thereby enabling species identification through sequencing. As a result, DNA-based sequencing is routinely employed for the detection of bacteria (It is important to note that sequencing instruments can only read the base sequences of DNA molecules and cannot directly read RNA molecules). In addition to bacteria, this method can also detect DNA viruses, fungi, and protozoa. Under specific circumstances, such as host genome integration or sample contamination, RNA viruses may also be detected. RNA sequencing is primarily used for the detection of RNA viruses. Furthermore, due to the presence of ribosomal RNA (rRNA) and messenger RNA (mRNA) in bacteria, this approach can also identify certain bacterial species. Statistical analysis from this study (Figure 7) demonstrates that the vast majority of bacteria, viruses, and fungi can be detected via DNA sequencing. For pathogens detected by both sequencing modalities, we cannot exclude the possibility that they were identified through the DNA sequencing modality. Notably, DNA viruses such as cytomegalovirus (CMV) were detected not only by DNA sequencing but also in the RNA sequencing modality. We hypothesize that this may be due to the transcription of RNA (primarily mRNA) from DNA viruses within infected cells, indicating a highly active state of viral replication. Conversely, RNA viruses like respiratory syncytial virus (RSV) were detected in the DNA sequencing modality, which may result from the conversion of viral RNA into complementary DNA (cDNA) during the sequencing library preparation process.

Furthermore, it is important to note that most pathogen detections involve polymicrobial infections—comprising bacteria, viruses, and fungi—rather than a single pathogen, a scenario commonly encountered in clinical practice. While this complexity highlights the need for comprehensive antimicrobial stewardship, careful attention must be given to potential adverse effects such as drug toxicity and disruption of microbiota balance associated with combination therapies. When interpreting the detection of multiple pathogens by mNGS, clinicians should prioritize the analysis of sequencing read counts for each organism and correlate these findings with the patient's specific clinical manifestations. This integrated approach allows for tailored antimicrobial therapy, including timely de-escalation or escalation of antibiotic regimens, as well as the potential addition of antiviral agents when supported by clinical and molecular evidence.

It should also be acknowledged that mNGS does not achieve 100% detection sensitivity, and false-negative results do occur. Nevertheless, these findings cannot completely rule out the presence of a pathogenic infection. Potential explanations include improper sampling techniques, suboptimal specimen preservation and handling, or the possibility of occult infections at other sites that may have gone undetected. When initial mNGS testing fails to identify a pathogen, the decision to retest should be guided by the response to empirical treatment. If the patient shows significant clinical improvement along with a marked decline in infection biomarkers, repeat mNGS testing may be unnecessary. Conversely, if clinical improvement is insufficient, a repeat mNGS assay is warranted.

In addition to false negatives, contamination of specimens—especially from sterile sites such as cerebrospinal fluid and peripheral blood—must be rigorously prevented. Strict aseptic technique during sample collection and handling is essential. Similarly, diagnostic laboratories must adhere to standardized protocols to ensure the high accuracy and reliability of mNGS testing.

## 5 Conclusion

This study evaluates the impact of sequencing modalities selection on pathogen detection efficiency, providing evidence-based insights for optimizing clinical practice. In developing countries where the cost of mNGS substantially exceeds that of routine tests such as complete blood counts or blood cultures, strategic selection of sequencing sequencing modalitiess—as opposed to universally defaulting to dual-sequencing modalities testing—can reduce financial burdens without compromising diagnostic accuracy.

mNGS is widely used in clinical practice (35–37), with current methodologies encompassing three sequencing modalities: DNA-only, RNA-only, and combined DNA/RNA. Our analysis revealed clear differences in sequencing modalities utilization and diagnostic performance. While DNA-sequencing modalities testing accounted for the majority of submissions (44.52%, 398/894), its positivity rate (50.75%, 202/398) did not surpass RNA-sequencing modalities (55.68%, 147/264) or combined-sequencing modalities testing (49.56%, 115/232), though these differences lacked statistical significance (*p* *>* *0.05*). Sample-specific performance varied markedly (*p* *<* *0.05*). Peripheral blood, the most frequently submitted specimen type (34.23%, 306/894), demonstrated limited diagnostic yield (43.14%, 132/306). In contrast, BALF achieved exceptional sensitivity (84.57%, 145/175), establishing it as the optimal choice for suspected respiratory infections. Combined DNA/RNA testing further enhanced BALF detection (97.33%, 73/75) and sputum analysis (82.35%, 14/17), outperforming single-sequencing modalities approaches. For systemic infections like sepsis—where rapid progression to intracranial involvement is common—concurrent blood and cerebrospinal fluid testing proved clinically valuable, with DNA-sequencing modalities testing showing superior performance in these specimen types. Therefore, in clinical work, we should make a preliminary judgment according to the clinical manifestations of patients, and send appropriate samples for examination. Especially for sputum and BALF samples, the positive rates of different sequencing modalitiess were also statistically different. (*p* *<* *0.05*). Pathogen profiling identified high-prevalence threats including *Human herpesvirus 5*, *Staphylococcus aureus*, *Klebsiella pneumoniae*, and *Escherichia coli*—organisms frequently associated with antimicrobial resistance. Beyond pathogen identification, mNGS demonstrated added utility in detecting antimicrobial resistance genes, enabling real-time monitoring of treatment efficacy (38, 39).

Chinese scholars have developed clinical guidelines for the application of mNGS in neonatal infections, which standardize its use in this population. The guidelines put forward under what circumstances should be tested by mNGS as much as possible, and what samples should be tested under different clinical manifestations (19).

## Data Availability

The datasets presented in this article are not readily available because the research data contains confidential patient information and therefore cannot be disclosed. Requests to access the datasets should be directed to the corresponding author.

## References

[1] Xu Y Zhu X Wang H Pan Z Li X Guo X Prevalence of major morbidities and outcome of all hospitalized neonates. A retrospective cohort study of huai'an neonatal survivals. J Matern Fetal Neonatal Med. (2022) 35(25):9800–10. 10.1080/14767058.2022.2054320 35341440

[2] Urban A Dyrda M. Mother and neonate suffering from COVID-19 infection. Is there any risk of vertical transmission? A case report. Ginekol Pol. (2021) 92(10):701–3. 10.5603/GP.a2021.0033 34541649

[3] Deng K Liang J Mu Y Liu Z Wang Y Li M Preterm births in China between 2012 and 2018: an observational study of more than 9 million women. Lancet Glob Health. (2021) 9(9):e1226–41. 10.1016/S2214-109X(21)00298-9 34416213 PMC8386289

[4] Horbar JD Greenberg LT Buzas JS Ehret DEY Soll RF Edwards EM. Trends in mortality and morbidities for infants born 24 to 28 weeks in the US: 1997–2021. Pediatrics. (2024) 153(1):e2023064153. 10.1542/peds.2023-064153 Erratum in: Pediatrics. 2024;153(5):e2024066036. doi: 10.1542/peds.2024-066036. 38053449

[5] Wu IH Tsai MH Lai MY Hsu LF Chiang MC Lien R Incidence, clinical features, and implications on outcomes of neonatal late-onset sepsis with concurrent infectious focus. BMC Infect Dis. (2017) 17(1):465. 10.1186/s12879-017-2574-7 28673280 PMC5496375

[6] Schrag SJ Farley MM Petit S Reingold A Weston EJ Pondo T Epidemiology of invasive early-onset neonatal sepsis, 2005 to 2014. Pediatrics. (2016) 138(6):e20162013. 10.1542/peds.2016-2013 27940705

[7] Xu H Ca D Zhou L. Diagnostic and prognostic value of PCT and RDW in premature infants with septicemia. Medicine (Baltimore). (2024) 103(7):e35725. 10.1097/MD.0000000000035725 38363932 PMC10869038

[8] Omran A Sobh H Abdalla MO El-Sharkawy S Rezk AR Khashana A. Salivary and serum interleukin-10, C-reactive protein, mean platelet volume, and CRP/MPV ratio in the diagnosis of late-onset neonatal sepsis in full-term neonates. J Immunol Res. (2021) 2021:4884537. 10.1155/2021/4884537 34676267 PMC8526251

[9] Fan G Guo Y Tang F Chen M Liao S Wang J. Determining the clinical characteristics, treatment strategies, and prognostic factors for *Mycoplasma pneumoniae* encephalitis in children: a multicenter study in China. J Clin Neurol. (2023) 19(4):402–9. 10.3988/jcn.2022.0328 37417436 PMC10329932

[10] Scheer CS Fuchs C Gründling M Vollmer M Bast J Bohnert JA Impact of antibiotic administration on blood culture positivity at the beginning of sepsis: a prospective clinical cohort study. Clin Microbiol Infect. (2019) 25(3):326–31. 10.1016/j.cmi.2018.05.016 29879482

[11] Hayder Hamad M Eidan Hadi M Ajam IK. Comparison between polymerase chain reaction and blood culture for diagnosis of neonatal sepsis. Arch Razi Inst. (2023) 78(1):221–6. 10.22092/ARI.2022.358608.2259 37312696 PMC10258275

[12] Hilt EE Ferrieri P. Next generation and other sequencing technologies in diagnostic microbiology and infectious diseases. Genes (Basel). (2022) 13(9):1566. 10.3390/genes13091566 36140733 PMC9498426

[13] Yang RL Qian GL Wu DW Miao JK Yang X Wu BQ A multicenter prospective study of mNGS-based newborn screening for monogenic genetic diseases in China. World J Pediatr. (2023) 19(7):663–73. 10.1007/s12519-022-00670-x 36847978 PMC10258179

[14] Shen G Li W Zhang Y Chen L. mNGS based newborn screening and comparative analysis with MS/MS. BMC Pediatr. (2024) 24(1):230. 10.1186/s12887-024-04718-x 38561707 PMC10985934

[15] Zhang XX Guo LY Liu LL Shen A Feng WY Huang WH The diagnostic value of metagenomic mNGS for identifying Streptococcus pneumoniae in paediatric bacterial meningitis. BMC Infect Dis. (2019) 19(1):495. 10.1186/s12879-019-4132-y 31164085 PMC6549306

[16] Wilson MR O'Donovan BD Gelfand JM Sample HA Chow FC Betjemann JP Chronic meningitis investigated via metagenomic next-generation sequencing. JAMA Neurol. (2018) 75(8):947–55. 10.1001/jamaneurol.2018.0463 Erratum in: JAMA Neurol. 2018 August 1;75(8):1028. doi: 10.1001/jamaneurol.2018.1554. 29710329 PMC5933460

[17] Chen W Zhou H. Etiological diagnosis and treatment value of mNGS in alveolar lavage fluid of children with severe pneumonia. Altern Ther Health Med. (2024) 30(7):65–71. 38702151

[18] Wang L Li S Qin J Tang T Hong J Tung TH Clinical diagnosis application of metagenomic next-generation sequencing of plasma in suspected sepsis. Infect Drug Resist. (2023) 16:891–901. 10.2147/IDR.S395700 36820080 PMC9938705

[19] Subspecialty Group of Neonatology, the Society of Pediatrics, Chinese Medical Association, Editorial Board, Chinese Journal of Pediatrics. Expert consensus on the application of metagenomics mNGS in neonatal infectious diseases. Zhonghua Er Ke Za Zhi. (2022) 60(6):516–21. Chinese. 10.3760/cma.j.cn112140-20220113-00046 35658355

[20] Duan H Li X Mei A Li P Liu Y Li X The diagnostic value of metagenomic next-generation sequencing in infectious diseases. BMC Infect Dis. (2021) 21(1):62. 10.1186/s12879-020-05746-5 33435894 PMC7805029

[21] Yang A Chen C Hu Y Zheng G Chen P Xie Z Application of metagenomic mNGS (mNGS) using BALF (BALF) in diagnosing pneumonia of children. Microbiol Spectr. (2022) 10(5):e0148822. 10.1128/spectrum.01488-22 36169415 PMC9603332

[22] Chen X Ding S Lei C Qin J Guo T Yang D Blood and BALF metagenomic mNGS in pneumonia. Can J Infect Dis Med Microbiol. (2020) 2020:6839103. 10.1155/2020/6839103 32879643 PMC7448216

[23] Wang L Zha P Wang Y Kong Y Su Y Dai L The value of macrogene second-generation sequencing in the diagnosis, guidance of drug use, and efficacy monitoring of infectious pneumonia in premature infants. Comput Math Methods Med. (2022) 2022:4398614. 10.1155/2022/4398614 36277011 PMC9581658

[24] Whitley RJ. Herpes Simplex virus infections of the central nervous system. Continuum (Minneap Minn). (2015) 21(6 Neuroinfectious Disease):1704–13. 10.1212/CON.0000000000000243 26633784

[25] Nhu NTK Phan MD Hancock SJ Peters KM Alvarez-Fraga L Forde BM High-risk *Escherichia coli* clones that cause neonatal meningitis and association with recrudescent infection. Elife. (2024) 12:RP91853. 10.7554/eLife.91853 38622998 PMC11021048

[26] Alarjani KM Almutairi AM AlQahtany FS Soundharrajan I. Methicillin and multidrug resistant pathogenic *Staphylococcus aureus* associated sepsis in hospitalized neonatal infections and antibiotic susceptibility. J Infect Public Health. (2021) 14(11):1630–4. 10.1016/j.jiph.2021.08.031 34624718

[27] Ma H Xu J Zhang Y Zhang R Wu J. Relevance and antimicrobial resistance profile of *Klebsiella pneumoniae* in neonatal sepsis. J Matern Fetal Neonatal Med. (2024) 37(1):2327828. 10.1080/14767058.2024.2327828 38471804

[28] Chang L Shi H Zhou W Hu ZQ Mu LY Su M Clinical characteristics and pathogens of invasive fungal infections in children. Zhongguo Dang Dai Er Ke Za Zhi. (2012) 14(12):933–7. Chinese. 23234781

[29] Zhu Y Gan M Ge M Dong X Yan G Zhou Q Diagnostic performance and clinical impact of metagenomic mNGS for pediatric infectious diseases. J Clin Microbiol. (2023) 61(6):e0011523. 10.1128/jcm.00115-23 Erratum in: J Clin Microbiol. 2023 November 21;61(11):e0115023. doi: 10.1128/jcm.01150-23. 37260394 PMC10281092

[30] Teoh T McNamara R Powell J O'Connell NH Dunne CP. A retrospective observational study of the impact of 16s and 18s ribosomal RNA PCR on antimicrobial treatment over seven years: a tertiary hospital experience. PLoS One. (2021) 16(10):e0258552. 10.1371/journal.pone.0258552 34637486 PMC8509882

[31] Han D Diao Z Lai H Han Y Xie J Zhang R Multilaboratory assessment of metagenomic next-generation sequencing for unbiased microbe detection. J Adv Res. (2021) 38:213–22. 10.1016/j.jare.2021.09.011 35572414 PMC9091723

[32] Wilson MR Sample HA Zorn KC Arevalo S Yu G Neuhaus J Clinical metagenomic sequencing for diagnosis of meningitis and encephalitis. N Engl J Med. (2019) 380(24):2327–40. 10.1056/NEJMoa1803396 31189036 PMC6764751

[33] Zhang R Wu Y Deng G Deng J. Value of sputum gram stain, sputum culture, and BALF gram stain in predicting single bacterial pathogen among children with community-acquired pneumonia. BMC Pulm Med. (2022) 22(1):427. 10.1186/s12890-022-02234-1 36402959 PMC9675245

[34] Garges HP Moody MA Cotten CM Smith PB Tiffany KF Lenfestey R Neonatal meningitis: what is the correlation among cerebrospinal fluid cultures, blood cultures, and cerebrospinal fluid parameters? Pediatrics. (2006) 117(4):1094–100. 10.1542/peds.2005-1132 16585303

[35] Zhang X Wang Y Pen D Liu J Zhou Q Wang Y Diagnosis of mixed infection and a primary immunodeficiency disease using mNGS: a case report. Front Cell Infect Microbiol. (2023) 13:1179090. 10.3389/fcimb.2023.1179090 37674579 PMC10477990

[36] Schmoch T Westhoff JH Decker SO Skarabis A Hoffmann GF Dohna-Schwake C mNGS diagnostics of bacteremia in pediatric sepsis. Medicine (Baltimore). (2021) 100(25):e26403. 10.1097/MD.0000000000026403 34160425 PMC8238315

[37] Okumura T Horiba K Tetsuka N Sato Y Sugiyama Y Haruta K mNGS-based detection of *Ureaplasma* in the gastric fluid of neonates with respiratory distress and chorioamnionitis. J Matern Fetal Neonatal Med. (2023) 36(1):2207113. 10.1080/14767058.2023.2207113 37150592

[38] Lin R Xing Z Liu X Chai Q Xin Z Huang M Performance of targeted mNGS in the detection of respiratory pathogens and antimicrobial resistance genes for children. J Med Microbiol. (2023) 72(11). 10.1099/jmm.0.001771 37910007

[39] Wu SH Xiao YX Hsiao HC Jou R. Development and assessment of a novel whole-gene-based targeted mNGS assay for detecting the susceptibility of mycobacterium tuberculosis to 14 drugs. Microbiol Spectr. (2022) 10(6):e0260522. 10.1128/spectrum.02605-22 36255328 PMC9769975

