# Correction: Channel selection of metagenomic next-generation sequencing in infants pathogen detection: a multicenter cross-sectional study

**DOI:** 10.3389/fped.2026.1835424

**Published:** 2026-05-28

**Authors:** Chengcheng Yang, Minxu Li, Shumei Yang, Jianer Pan, Yue Ding, Jie Yang

**Affiliations:** 1 Department of Neonatology, Nanfang Hospital, Southern Medical University, Guangzhou, China; 2 Department of Neonatology, Dongguan Women and Children Hospital, Dongguan, Guangdong, China; 3 Department of Neonatology, Guangdong Women and Children Hospital, Guangzhou, China; 4 Department of Neonatology, Jiangmen Women and Children Hospital, Jiangmen, Guangdong, China

**Keywords:** channel, infectious diseases, MNGs, neonate, pathogene

Author Yang Shumei was erroneously assigned to **affiliation** “Department of Neonatology, Nanfang Hospital, Southern Medical University, Guangzhou, China”. This affiliation has now been removed for author Yang Shumei. **Affiliation** “Department of Neonatology, Guangdong Women and Children Hospital, Guangzhou, China” was omitted for author Yang Shumei. This affiliation has now been added for author Yang Shumei.

In the **abstract**, there was a clerical error during data entry. This has been corrected to read:

“Sputum samples showed a 53.7% positivity rate (87/162), increasing to 82.35% (14/17) with dual-channel detection”.

A correction has been made to **section 3.3.1**, *paragraph 25* as there was a clerical error during data entry:

“The total number of samples sent for testing was 894, with 464 positive cases detected, resulting in an overall positive rate of 51.90% (464/894)./In contrast, sputum specimens (*n* = 162) demonstrated profound combined-sequencing modalities superiority, achieving an 82.35% positivity rate (14/17) versus DNA-only (58.02%, 47/81) and RNA-only (40.62%, 26/64) (*p* = 0.005).]”

An incorrect **Funding** statement was provided. The correct funders are “National Natural Science Foundation of China”, and “National Key Research and Development Program of China”. The correct funding statement reads:

“The author(s) declare that financial support was received for the research and/or publication of this article. This work was supported by the National Natural Science Foundation of China (82171714). Author JY was supported by the National Key Research and Development Program of China (2021YFC2701).”

The original version of this article has been updated.

